# Ensemble Modeling of Multiple Physical Indicators to Dynamically Phenotype Autism Spectrum Disorder

**DOI:** 10.3390/a18120764

**Published:** 2025-12-02

**Authors:** Marie Amale Huynh, Aaron Kline, Saimourya Surabhi, Kaitlyn Dunlap, Onur Cezmi Mutlu, Mohammadmahdi Honarmand, Parnian Azizian, Peter Washington, Dennis P. Wall

**Affiliations:** 1 Department of Biomedical Data Science and Department of Pediatrics, Stanford University, Stanford, CA 94305, USA; 2 Division of Clinical Informatics & Digital Transformation, Department of Medicine, University of California, San Francisco, CA 94143, USA

**Keywords:** autism, video-based phenotyping, data fusion

## Abstract

Early detection of Autism Spectrum Disorder (ASD), a neurodevelopmental condition characterized by social communication challenges, is essential for timely intervention. Naturalistic home videos collected via mobile applications offer scalable opportunities for digital diagnostics. We leveraged GuessWhat, a mobile game designed to engage parents and children, which has generated over 3000 structured videos from 382 children. From this collection, we curated a final analytic sample of 688 feature-rich videos centered on a single dyad, enabling more consistent modeling. We developed a two-step pipeline: (1) filtering to isolate high-quality videos, and (2) feature engineering to extract interpretable behavioral signals. Unimodal LSTM-based models trained on eye gaze, head position, and facial expression achieved test AUCs of 86% (95% CI: 0.79–0.92), 78% (95% CI: 0.69–0.86), and 67% (95% CI: 0.55–0.78), respectively. Late-stage fusion of unimodal outputs significantly improved predictive performance, yielding a test AUC of 90% (95% CI: 0.84–0.95). Our findings demonstrate the complementary value of distinct behavioral channels and support the feasibility of using mobile-captured videos for detecting clinically relevant signals. While further work is needed to improve generalizability and inclusivity, this study highlights the promise of real-time, scalable autism phenotyping for early interventions.

## 1. Introduction

Autism Spectrum Disorder (ASD, or autism) is a complex neurodevelopmental condition that affects approximately 1 in 36 children across diverse ethnic, racial, and socioeconomic backgrounds, highlighting its widespread impact and the need for comprehensive strategies to address it [1]. Children with autism face significant challenges in communication, social interactions, repetitive behaviors, and restricted interests, often leading to profound difficulties in everyday functioning and development [2,3]. The impact of autism extends beyond individual health, imposing a substantial economic burden, with an estimated lifetime social cost of approximately USD 3.6 million per affected individual [4].

Early interventions are crucial, as the developmental gap between children with autism and neurotypical (NT) children widens over time [5]. Early diagnosis can lead to improved health outcomes; however, despite the potential for reliable diagnosis as early as 16–24 months, the average age of diagnosis is 4.9 years [6,7]. Diagnostic processes typically involve long waitlists and assessments, resulting in an average delay of two years [8]. Current diagnoses rely on in-person behavioral assessments, which are costly and subjective, lacking definitive medical tests or biomarkers [9]. This subjective process introduces variability and the potential for misdiagnosis influenced by clinician experience, training, and social biases [1,10–13].

Digital phenotyping, leveraging naturalistic home videos and affordable smartphones, offers a promising approach for faster, more objective diagnosis of autism and other developmental conditions. GuessWhat [14] is a gamified mobile application designed to facilitate the collection of videos enriched with behaviors relevant to autism diagnosis [15] while providing a set of tools that can lead to improved social communication skills for children with autism [16]. Here, we explore the development of multimodal deep learning models that integrate multiple behavioral modalities (eye, face, and head data) to facilitate comprehensive, automated autism diagnosis.

## 2. Related Works

### 2.1. Digital Phenotyping of Autism

Computer vision tools have shown promise in identifying multiple phenotypes—such as emotion, eye movement, and posture tracking—in children through video analysis [17–19]. However, these tools often lack structured data on children with autism, and predictive models using individual phenotypes have rarely been assessed for fairness or integrated into ensemble models.

Modalities such as eye gaze [20–25], head movement [17,26,27], facial expression [24,28–31], finger movement [32–34], and vocalization [35,36] have proven to be valuable for automatically identifying autism. Nonetheless, challenges related to data variability, generalizability across diverse populations, and integration with other diagnostic measures remain.

Building upon these foundational works, GuessWhat provides structured data to address the lack of well-organized datasets for children with autism. The design of GuessWhat was informed by co-design with families of children with autism and validated in prior feasibility and clinical studies to ensure developmental appropriateness, user engagement, and consistent capture of behavioral features across participants [24,35,37,38]. We utilize deep time-series models, such as long short-term memory (LSTM) and gated recurrent units (GRUs), to analyze eye gaze, face, and head features in a multimodal fashion to predict autism. Our filtering and feature engineering pipelines address key challenges such as data variability and generalizability, improving the quality of the extracted features. We also evaluate fairness with respect to age and gender to ensure that our models generalize well across diverse populations. Ultimately, by integrating multiple phenotypes into a single model, we overcome the limitations of single-modality models, thereby improving predictive accuracy and paving the way for a more comprehensive understanding of autism diagnosis.

### 2.2. Data Fusion for ASD Classification

Recent studies have focused on the potential of data fusion for improving autism classification performance. Perochon et al. [39] recently evaluated a digital autism screening application—Sensetoknow—administered during pediatric well-child visits for children aged 17–36 months. Using XGBoost, they integrated multiple digital phenotypes, achieving an AUROC of 90%, sensitivity of 87.8%, specificity of 80.8%, negative predictive value of 97.8%, and positive predictive value of 40.6%. This app was later validated in another study, where it was remotely administered by caregivers at home using smartphones or tablets, demonstrating similarly high accuracy for autism detection [40]. These findings underscore the potential of combining data sources using tree-based machine learning methods to improve diagnostic outcomes.

Building on the fusion of multiple data types, Han et al. [41] integrated neurophysiological and behavioral data by developing a multimodal diagnostic framework for autism that combines electroencephalogram and eye-tracking data. Their two-step feature learning and fusion model, based on a stacked denoising autoencoder, outperformed unimodal approaches, demonstrating the advantages of combining data types. Wadhera [42] further expanded this approach by incorporating laptop-performance tool data and applying a kernel-based discriminant correlation analysis, which reduced feature redundancy and achieved 92% accuracy in autism classification. These findings highlight the effectiveness of multimodal systems in identifying influential features and clustering individuals into more homogeneous subgroups for the diagnosis of autism.

Our research uses mobile videos captured during at-home gameplay for scalable and dynamic autism data collection [43,44]. By continuously monitoring authentic behavioral data, we aim to facilitate earlier and more accurate diagnoses. We differentiate from these prior works by exploring the utility of multimodal deep learning and feature engineering. By analyzing such video data in real-world settings, we aim to bridge the gap between clinical practice and everyday environments, offering a practical solution for widespread autism screening and diagnosis.

## 3. Materials and Methods

### 3.1. Dataset

#### 3.1.1. Data Collection and Type

**Figure 1.**
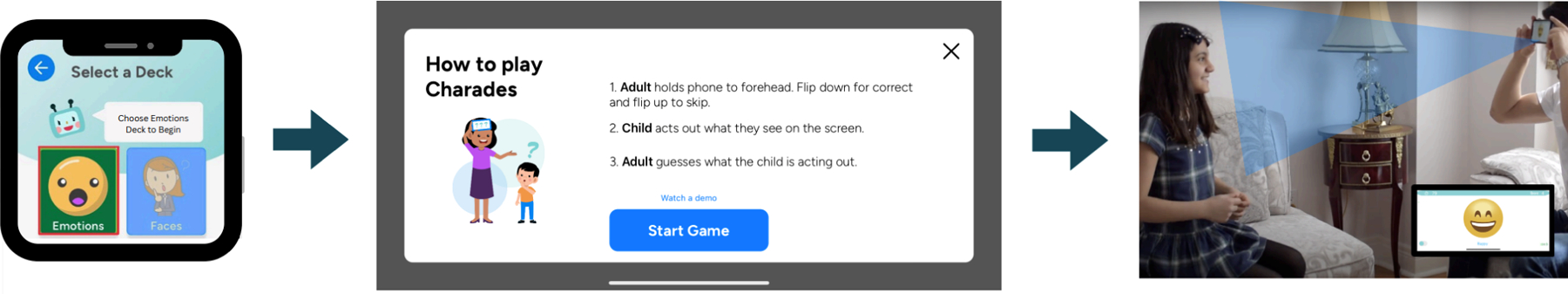
Illustration of the mobile game GuessWhat. GuessWhat is a charades-style game available for any smartphone device. In a typical game session, the parent holds the smartphone to their forehead and tries to guess the emotion mimicked by the child in response to the prompt shown on the phone’s screen. Each game session typically lasts 90 s.

Data were collected through the mobile game GuessWhat [45], designed to facilitate structured, charades-style interactions between parent and child. As illustrated in Figure 1, the parent holds the phone to their forehead, and the phone’s front camera captures the child acting out prompts (e.g., “happy,” “sad,” “elephant,” “baseball”), with parents opting to share videos after each session, stored securely on HIPAA-compliant servers. A Stanford-approved Video/Photo Release and informed consent were obtained from legal guardians for the use of the images presented in the figures in this manuscript. The study protocol and app were reviewed and approved by the Stanford Institutional Review Board (IRB) in accordance with Stanford, California, United States, and international research guidelines, including the Declaration of Helsinki, prior to the initiation of any study procedures. Informed consent was obtained from parents/legal guardians through the app before study participation. Recruitment, focusing primarily on children with autism, used crowd-sourcing, email, online ads, and the presence of the app store, resulting in a higher percentage of children with ASD than children without ASD (no-ASD). To participate, families were required to be able to read and understand the available translations of the mobile game, have access to a compatible iOS or Android device with internet connectivity, and be an adult parent or guardian of a child aged 1.5 to 17 years old. To safeguard against the potential for self-reporting bias, we required the caregiver to confirm that their child’s autism diagnosis came from a formal medical assessment [46] and asked parents to provide answers to a set of clinical instruments that included the Vineland Adaptive Behavior Scales-Parent Report [47], Social Responsiveness Scale [48], Mobile Autism Risk Assessment [49], Social Communication Questionnaire [50], and Autism Symptom Dimensions Questionnaire [51] to establish a preliminary confirmation of autism diagnosis (or lack thereof); subsequently, each video provided was independently examined in a blinded fashion by a clinician with expertise in autism diagnosis to provide a clinical diagnostic impression. We required the parent-reported diagnosis to match the clinician’s observational diagnosis, to include the video for analysis. This rigorous two-point consensus was used to ensure the ground truth while minimizing the parental burden of participation. To date, GuessWhat has collected over 3000 videos from 382 children, aged 1.5 to 17, with and without autism. We refer to this collection as the GuessWhat Dataset. For conciseness, we report detailed demographic breakdowns by split in Section 3.4.1 Data Splitting and Task Definition. While the consent process allows for participation beginning at 16 months, few videos were collected in the 16–24-month range, and those did not meet the inclusion criteria. Future efforts should aim to capture more data in this early window to support even earlier detection and intervention. That said, given that the average age of autism diagnosis in the U.S. is around 5 years, focusing on children aged 2 to 5 remains clinically relevant and impactful.

#### 3.1.2. Filtering Pipeline

**Figure 2.**
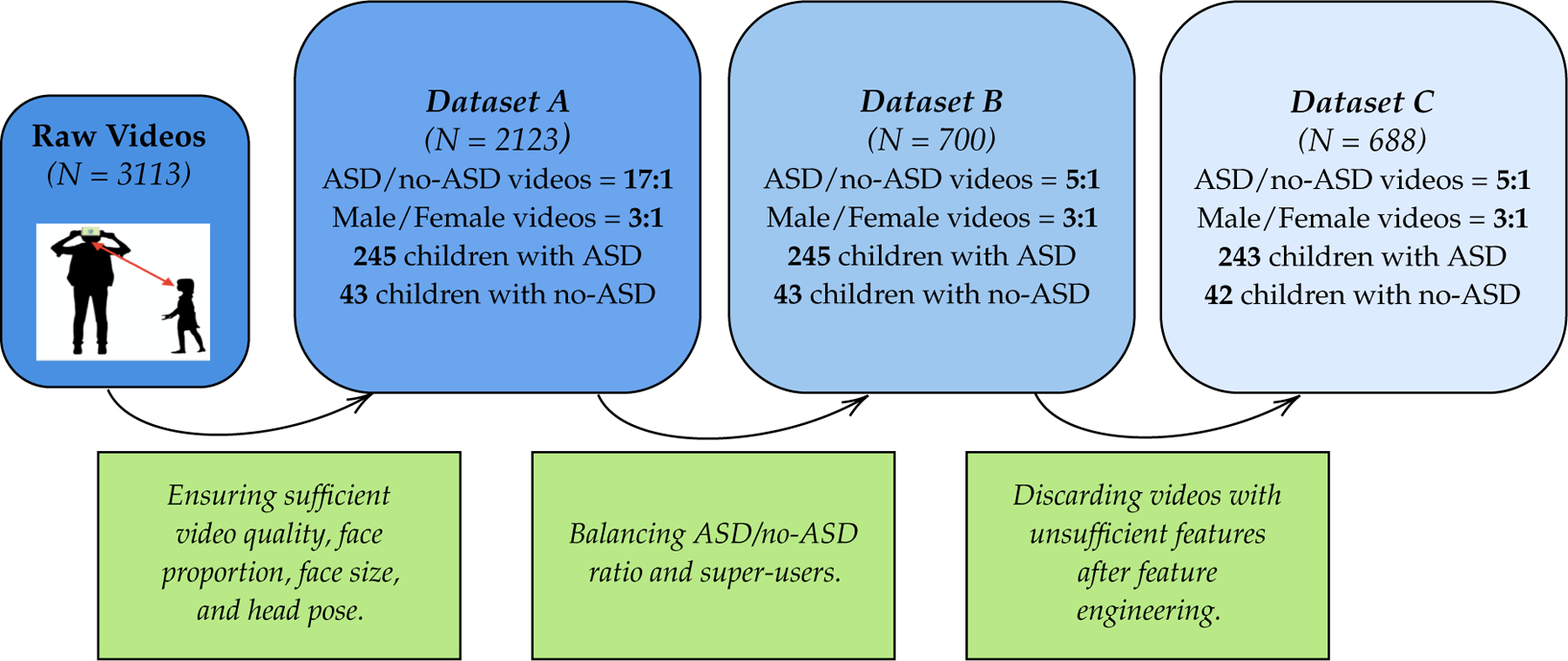
Key filtering steps, leading to 3 subsets containing increasingly filtered portions of the GuessWhat dataset.

The instability of home videos, often caused by handheld cameras or movement, can create noise and data drift that reduce the models’ performance. Furthermore, some videos may be feature-poor, or the child of interest may be too far away to extract the desired features, etc. Rigorous filtering and feature engineering are necessary to address these limitations and construct a minimally viable training set for our task. Our filtering pipeline can be summarized in three steps (Figure 2). The criteria for including videos from the overall GuessWhat Dataset in our final filtered dataset were as follows: (1) they must be of high quality, suitable for feature extraction; and (2) each video must focus on the child of interest. To ensure these criteria, we filtered our videos using the Amazon Rekognition Video API for face detection [52], which provides estimates for sharpness, brightness, head pose, facial landmarks, and face size for each video. For the first criterion, we selected videos that guarantee clarity through sharpness and brightness metrics, feature a sufficiently large face for detecting eye-gazing features, have the eyes open for more than 70% of the video’s duration, and maintain the face predominantly facing the camera. To meet the second criterion, we selected videos where the face is proportionally large enough for reliable feature extraction and where the presence of multiple faces is minimal, ensuring that the focus remains on a single child. While the game’s format ensures that the parent playing will not enter the frame during recording sessions, there are instances where the child’s siblings or friends join them onscreen. Thus, we currently adopt a strict approach, discarding videos with a high proportion of frames containing multiple faces, so as to avoid introducing uncertainty about which child the diagnosis refers to. However, a future step will involve incorporating child recognition techniques, allowing us to identify and focus on the child of interest, preventing the loss of potentially useful data. All thresholds are summarized in Appendix A (Table A1). This filtered dataset, referred to as ‘Dataset A’, includes 2123 videos featuring 288 children.

#### 3.1.3. Bias and Imbalances

**Figure 3.**
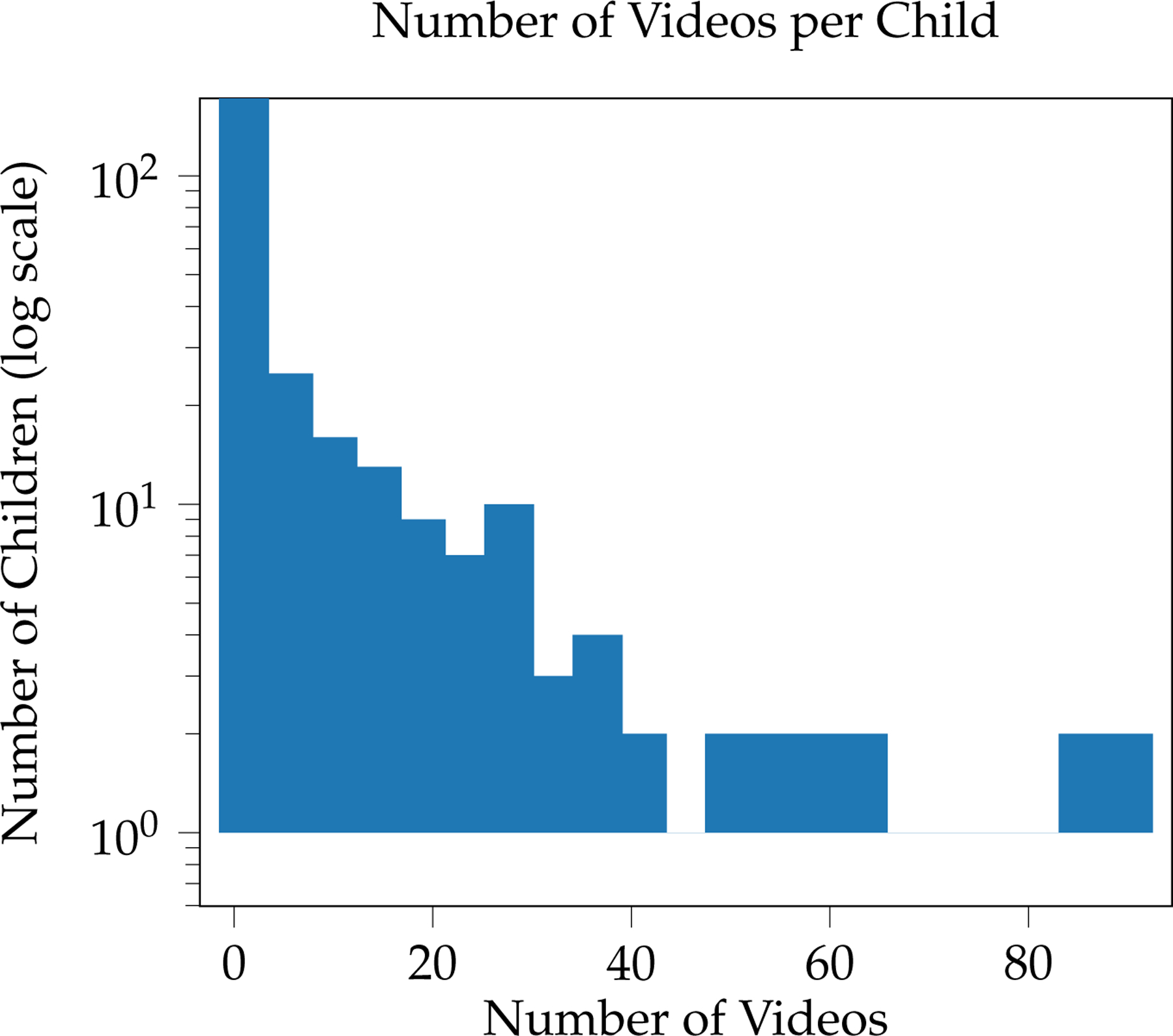
Presence of ‘superusers’. Some children dominate the data, with dozens of videos.

Our dataset contains a significant imbalance with respect to the ASD and no-ASD classes, with an ASD-to-no-ASD ratio of 17:1 (Figure 2). Furthermore, some users in the ASD class are more represented than others due to more gameplay (Figure 3). With only 116 videos for no-ASD children, we manually inspected each one to filter out low-quality or non-compliant videos. During this review, 7 videos were excluded due to issues such as the parent playing, the child holding the phone, or siblings appearing sequentially in the same video.

To reduce bias towards a particular child and mitigate the imbalance between ASD and no-ASD, we under-sampled the ASD class by retaining at most two videos per child, prioritizing those with the highest quality (based on sharpness and brightness). Retaining all superusers—who are disproportionately represented in the ASD group—biases the learning process toward a small number of specific individuals, leading to overfitting and near-random generalization performance on unseen children. For no-ASD children (the minority class), we retained all available videos. This balanced dataset, nicknamed ‘Dataset B,’ includes 700 videos (Figure 2). We also tested oversampling no-ASD and adding loss weights to manage class imbalance; however, these strategies did not yield satisfactory model performance, as illustrated in Appendix A (Table A5).

### 3.2. Feature Extraction

**Figure 4.**
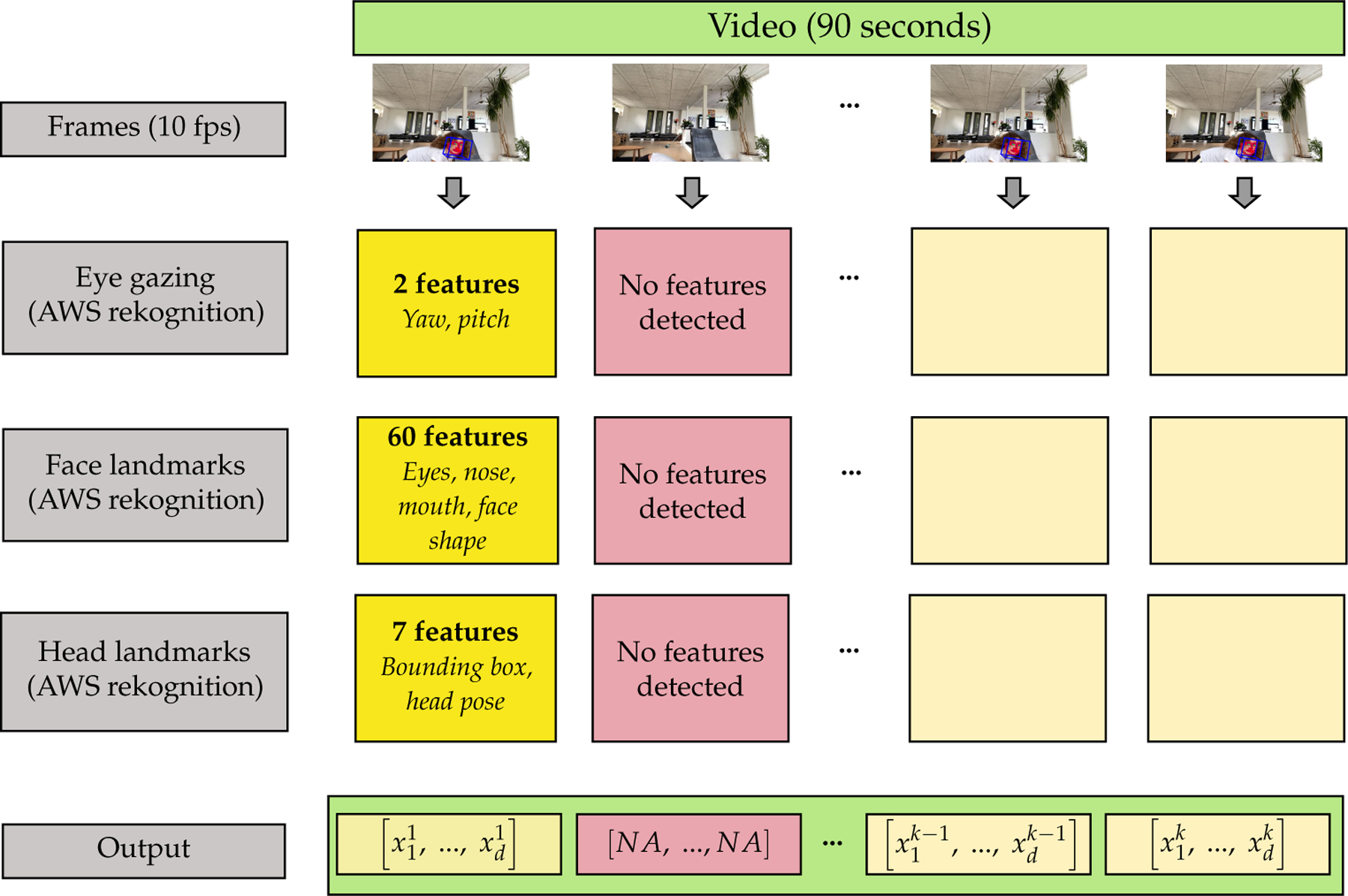
Feature extraction scheme.

GuessWhat videos have an average frame rate of 28 frames per second (fps) and an average length of 90 seconds (s). We downsampled the Dataset B videos to 10 fps and utilized AWS Rekognition [52] to extract frame-level eye gaze, head, and face features (Figure 4). Feature extraction for a 90 s video sampled at 10 fps costs approximately USD 0.90 per sample. Across the 628 videos processed in this study, this represented a substantial but manageable computational expense. Although this was the most reliable option available to us at the time, newer and more affordable alternatives now offer comparable accuracy and are expected to operate in real time, improving the feasibility of large-scale or mobile deployment. After extraction, we discarded any video with fewer than 15 s of extracted features and obtained a high-quality ‘Dataset C’ comprising 688 videos (Figure 2).

### 3.3. Data Preprocessing

**Figure 5.**
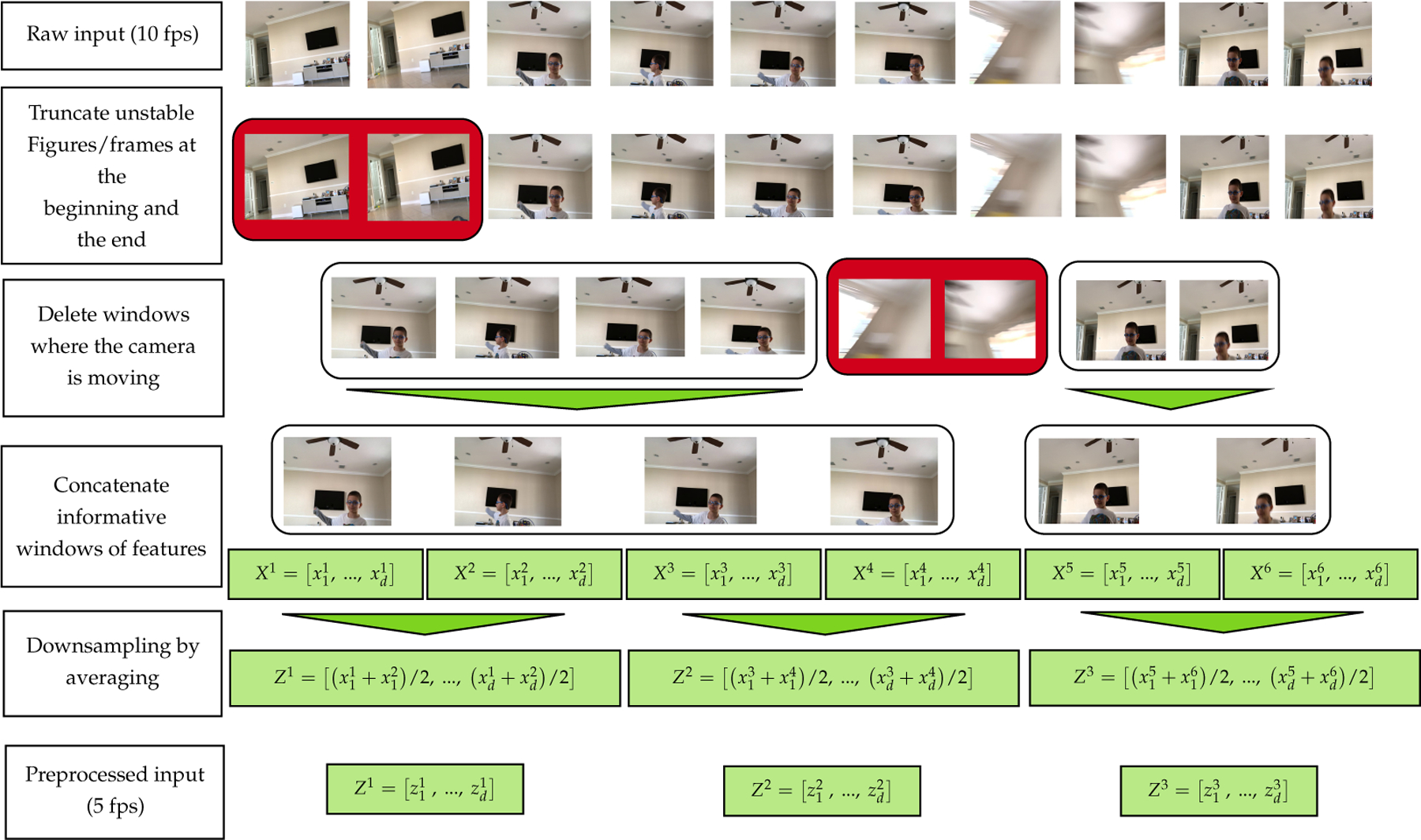
Key feature engineering steps.

Mobile data is inherently noisy, requiring robust feature engineering to extract informative sequences for learning. Our feature engineering pipeline, depicted in Figure 5, addresses several challenges.

At the start and end of videos, our features of interest are often missing due to camera stabilization, game initiation, or game completion with the child. To mitigate this, we truncate frames where no face is detected. Within the videos, we identify periods of missing data. Missing eye-gaze data occurs when the child’s eyes are closed, the child is not facing the camera, or the camera angle is misaligned. These instances can be informative, reflecting the child’s interaction challenges or meaningful movements. Conversely, periods without face detection or with poorly centered cameras provide no useful information. To balance these factors, we allow for brief periods of missing face data (up to 2 s) but discard segments with prolonged absence, as they provide little modeling signal. We exclude these uninformative windows and concatenate adjacent informative segments to enhance feature continuity. Our procedures for truncation, window creation, and segment concatenation are detailed in Appendix A (Algorithms A1, A2, and A3, respectively). To reduce the input length, we averaged features every two frames, for an effective frame rate of 5 fps (Figure 5). This downsampling strategy aligns with prior work, which shows that a frame rate of 5 fps is sufficient to preserve key behavioral dynamics in mobile video-based developmental assessments [15,53,54]. We then normalized the features using min–max normalization and replaced missing frames with vectors of −1, allowing us to explicitly encode missingness as part of the temporal data structure.

### 3.4 Model Training

#### 3.4.1. Data Splitting and Task Definition

The resulting dataset was split at the child level (in opposition to the video level) into training, validation, and test sets to prevent data leakage, especially for children with multiple videos. To ensure fairness and representativeness, we stratified the splits by age group (2–4, 5–8, 9–12) and gender (male, female), as summarized in Table 1. Corresponding video-level statistics are provided in Table 2. We chose this stratified child-level split over k-fold cross-validation because enforcing both child-level independence and balanced demographic distributions across multiple folds is infeasible given the limited number of children in certain age–gender groups. The input of our models consists of a sequence of k frames, where each frame contains a feature vector of size d (d being the dimension of features for a given modality: *d* ∈ {2, 7, 60} for the eye-gazing, head, and face modalities, respectively). Hence, our input is a multivariate time series denoted as [*X*^1^, …, *X*^*k*^], where $X^{i}=\left[x_{1}^{i},\ldots ,x_{d}^{i}\right]$, and our output is a binary label Y, where *Y* ∈{0, 1} (0: no-ASD, 1: ASD).

#### 3.4.2. Unimodal Deep Time-Series Models

We trained LSTM and GRU models in PyTorch v1.9.0 (CUDA 11.1) [55] for binary classification, using three input modalities: eye gaze, head pose, and facial expressions. These models accommodate variable-length input sequences, making them well suited to our data. Each model consists of *n* recurrent layers (*n* ∈ [4, 8]), each with hidden size *h* ∈ {16, 32, 64}. These parameters were tuned as part of the hyperparameter search detailed in Appendix A (Table A2). To address class imbalance, we experimented with focal loss [56], but we observed similar performance to that of standard binary cross-entropy. For training stability, we applied early stopping based on validation loss with a patience of 3 epochs and a minimum delta of 0.001.

#### 3.4.3. Fusion and Ensemble Techniques

To enhance predictive performance, we explored multimodal learning through three fusion strategies. In late fusion, we experimented with both (1) averaging the predicted probabilities of unimodal models and (2) concatenating their logit outputs and passing them through a linear layer for final prediction. In intermediate fusion, we (3) concatenated the final hidden states of pretrained unimodal models and fed them into a multi-layer perceptron. All fusion models were trained using binary cross-entropy loss with class weights. We evaluated each fusion strategy across all bimodal combinations and the full trimodal setting. A schematic overview of these approaches is provided in (Appendix A, Figure A3). Finally, for completeness, we also report the performance of a direct early-fusion baseline based on feature concatenation, although this approach is inherently less well suited to our imbalanced feature dimensionalities and the limited size of our dataset.

#### 3.4.4. Hyperparameter Optimization

For hyperparameter optimization, we utilized the Optuna Framework [57], which employs a Bayesian optimization approach using a Tree-structured Parzen Estimator (TPE) to fine-tune our models across various tasks. We selected the macro-averaged (MA) F1 score on the validation set as the optimization objective, because it jointly balances precision and recall while equally weighting all classes, unlike the weighted average, which is dominated by the majority ASD class. The hyperparameter search space is detailed in Appendix A (Table A2). For each model configuration, we ran 40 trials to identify the optimal parameters. The best-performing hyperparameter settings for each model are also reported in Appendix A (Tables A4, A6 and A7).

## 4. Results

### 4.1. Effects of Feature Engineering

Table 3 shows the impact of feature engineering on the performance of the unimodal models by comparing their AUC and macro-averaged (MA) F1 scores before and after applying the pipeline described in Figure 5. The eye model shows the most significant improvement. The head model also shows improvement, although its F1 score (MA) decreases slightly, indicating some trade-offs. The face model exhibits minimal improvement, with its AUC slightly increasing and the F1 score (MA) decreasing, showing limited effectiveness of feature engineering.

### 4.2. Performance Comparison of Our Models

**Figure 6.**
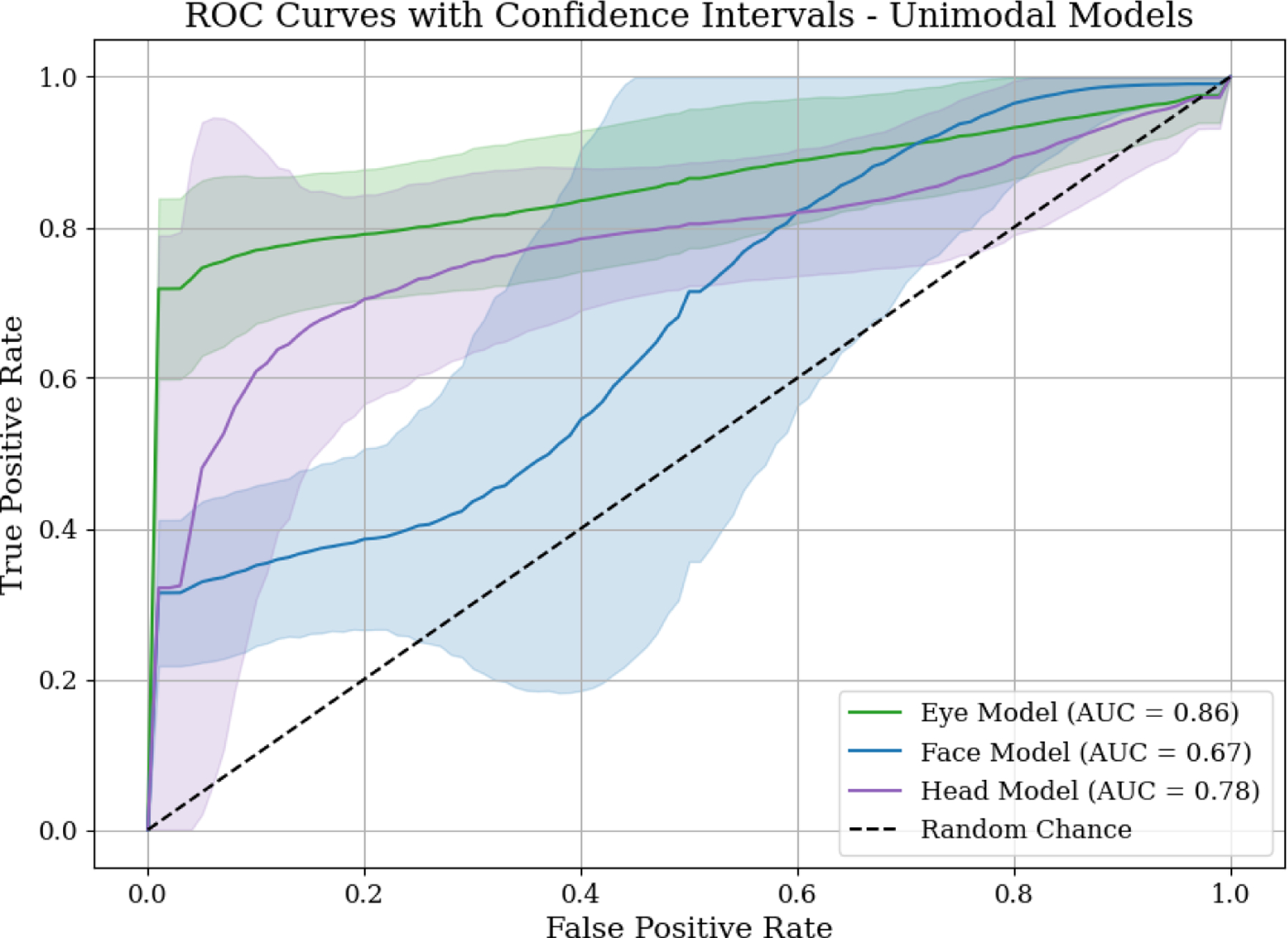
ROC curves of the unimodal models.

**Figure A1.**
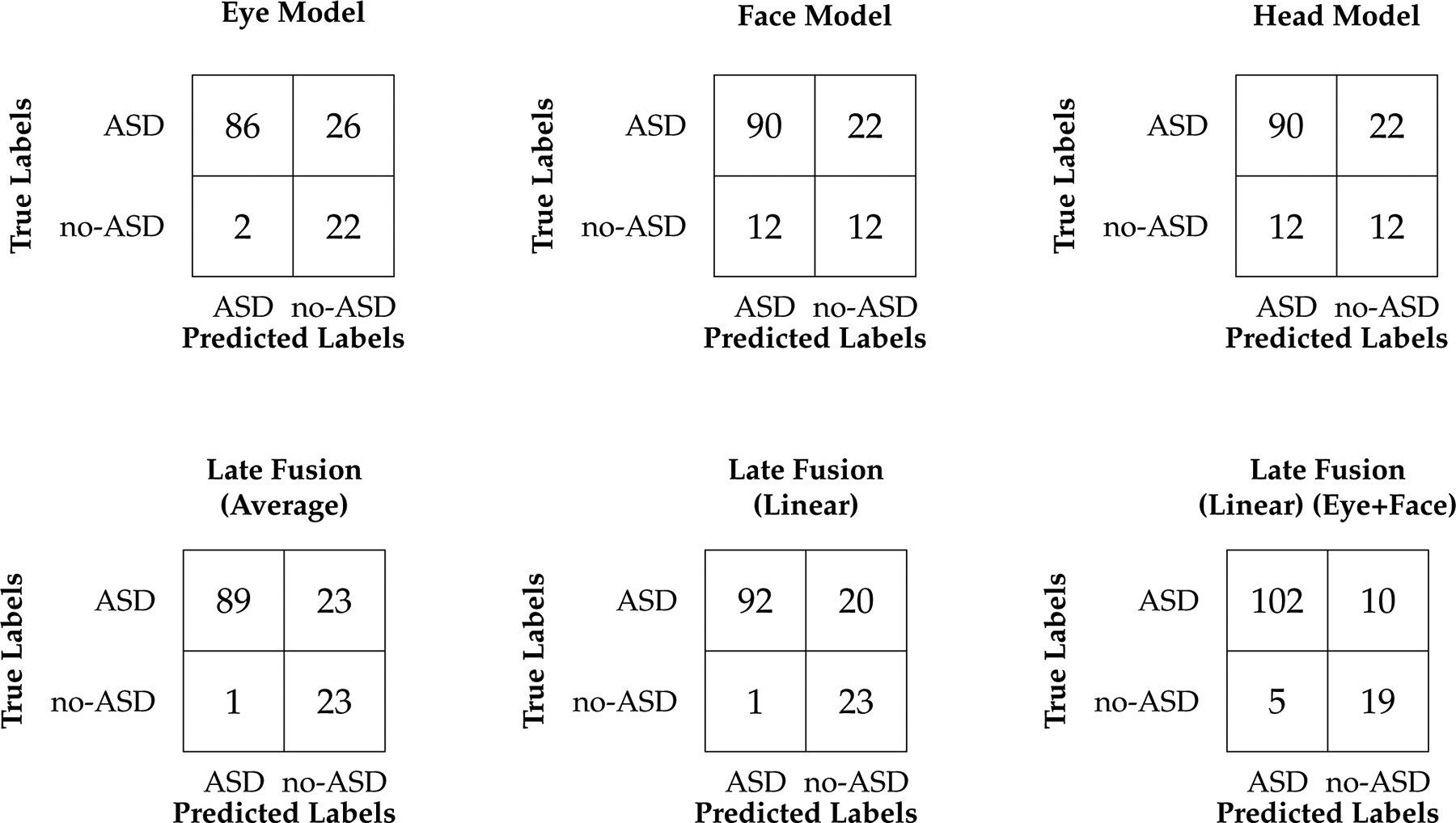
Confusion matrices for three unimodal models and three multimodal models: late fusion (average), late fusion (linear), and late fusion (linear) with eye+face inputs. Rows correspond to true labels and columns to predicted labels. A classification threshold of 0.5 was used.

**Figure A3.**
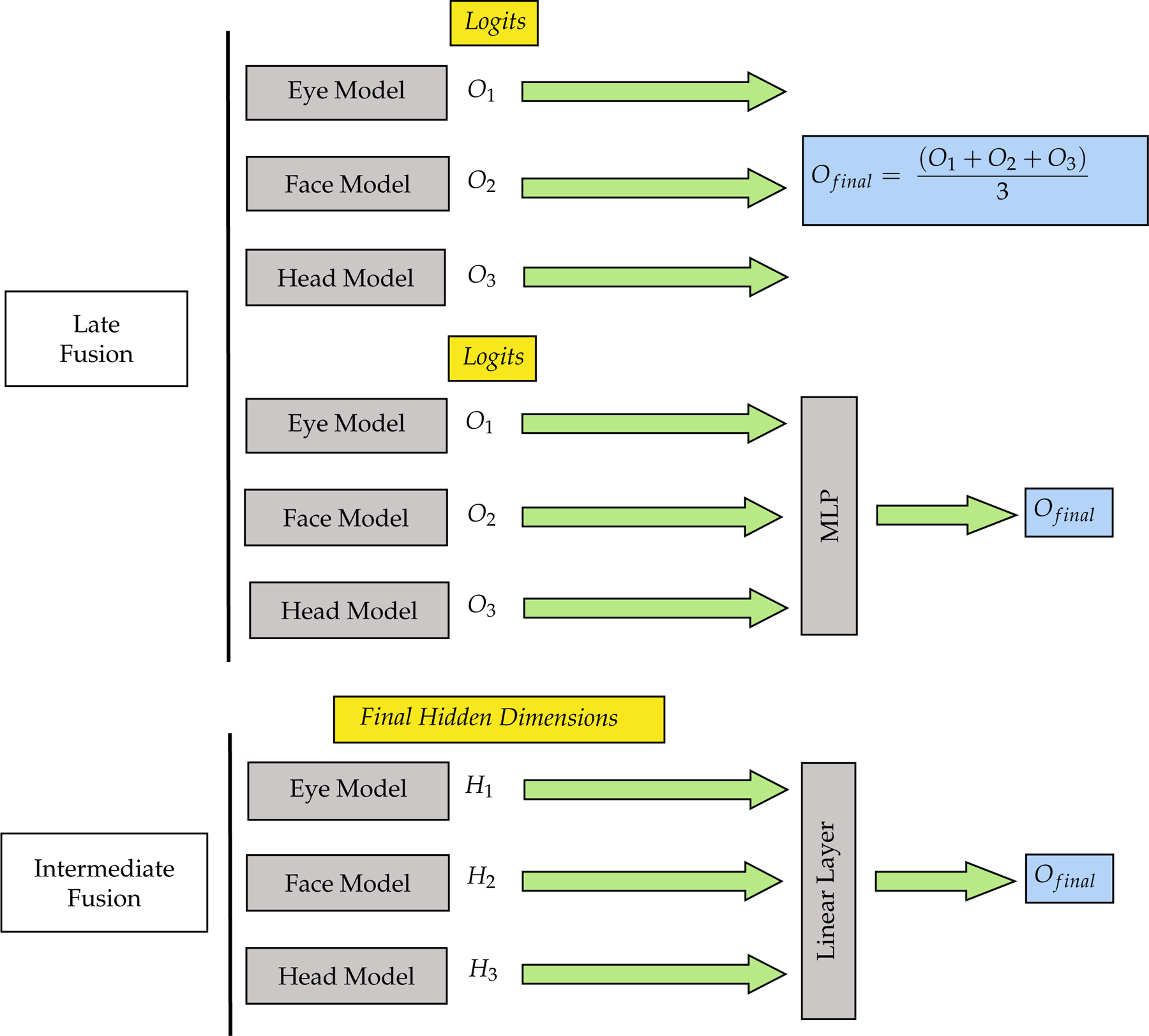
Late and intermediate fusion schemes.

We considered seven distinct input configurations: three unimodal models (eye gaze, head pose, facial expressions), three bimodal combinations, and one trimodal fusion. For each unique input setting and fusion approach, we selected the best model configuration based on the validation macro-averaged F1 score from 40 hyperparameter optimization trials, as described in the Section 3.4.4, Hyperparameter Optimization. The best-performing hyperparameter settings for each model are reported in Appendix A (Table A4). The eye and head models achieved strong predictive power, with test AUCs of 0.86 and 0.78, respectively (Figure 6). The face model had moderate predictive power, with a test AUC of 0.67. Table 4 summarizes the performance metrics across all unimodal models. Confidence intervals were estimated via bootstrapping: we resampled the test set 1000 times and computed the performance metrics on each sample. The 2.5th and 97.5th percentiles define the bounds of the 95% confidence intervals. Confusion matrices for all models are provided in Appendix A (Figure A1).

**Figure 7.**
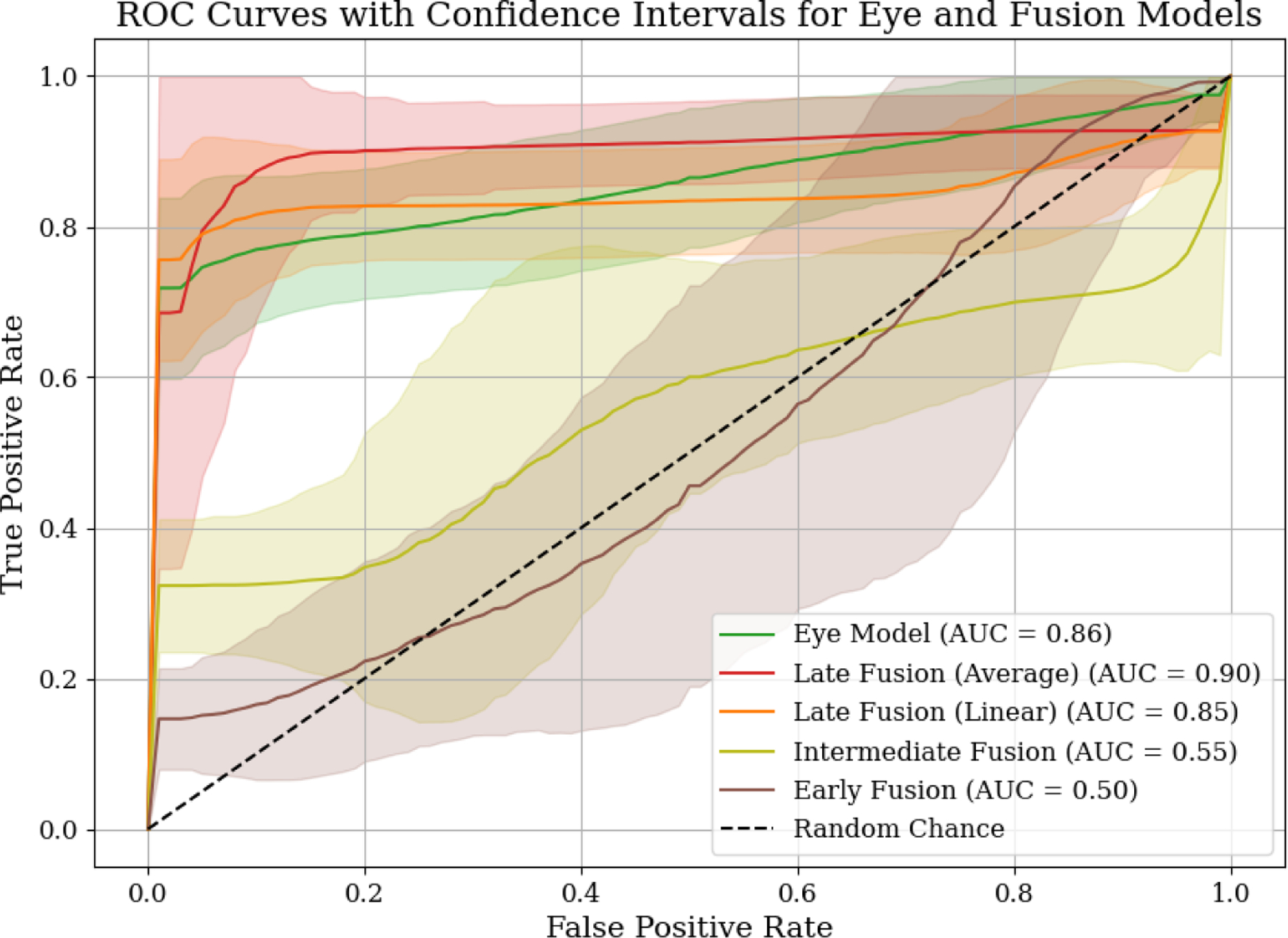
ROC curves of the eye and fusion models.

The eye model outperforms the face and head models, with narrower confidence intervals indicating more consistent performance. Its high F1 score (MA) reflects balanced precision and recall, making it the strongest unimodal model. This is further supported by the confusion matrix (Appendix A, Figure A1), which shows that the eye model achieves high precision for no-ASD (22/24 correctly classified) and recall for ASD (86/112). Integrating all three modalities further improves the predictive power: the late fusion models—particularly the averaging method (AUC: 0.90) and the linear combination method (AUC: 0.84)—achieve strong results (Figure 7). These models also improve no-ASD classification, correctly identifying 23 out of 24 no-ASD instances while maintaining high ASD recall. In contrast, the intermediate fusion and early fusion models perform substantially worse, with a test AUC of 0.55 and 0.50, respectively.

**Figure 8.**
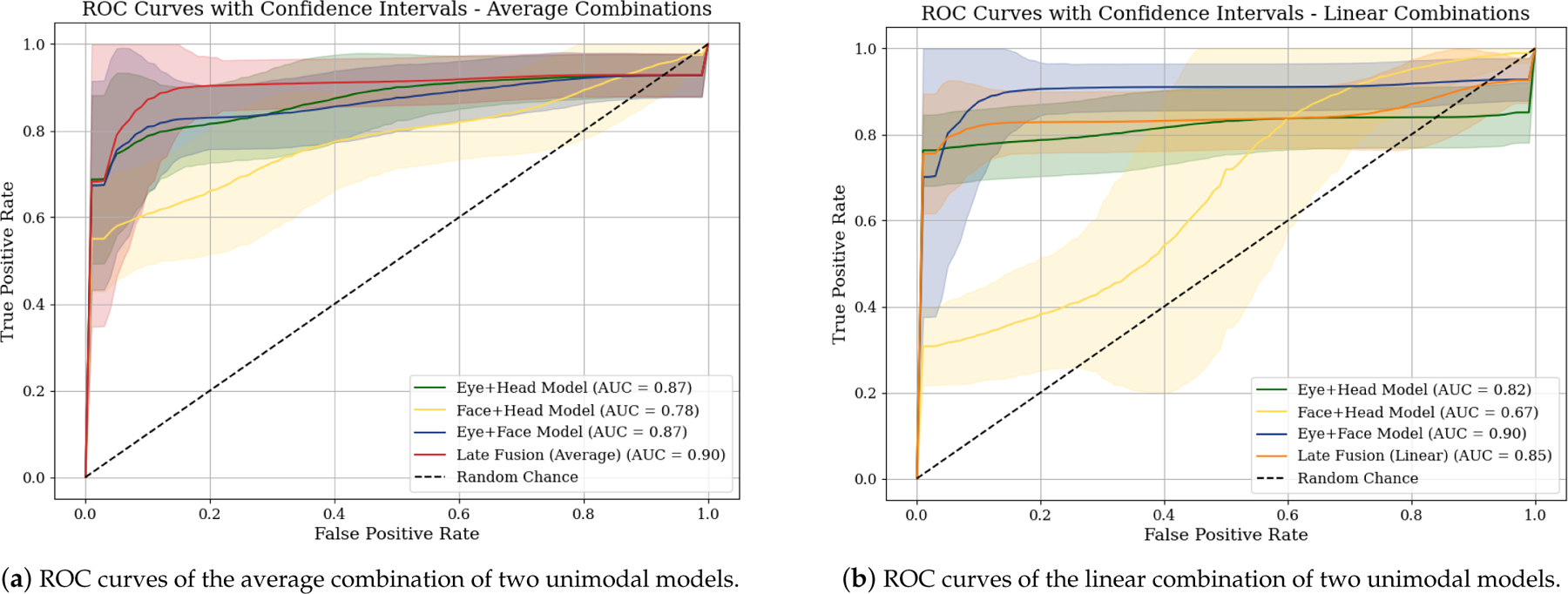
ROC curve results for fusion strategies: (**a**) average combination of two unimodal models, and (**b**) linear combination of two unimodal models.

Two-by-two feature combinations also show strong results. For late fusion by averaging, the eye+head and eye+face combinations each achieve an AUC score of 0.87, while the face+head model achieves an AUC score of 0.78 (Figure 8a). With a linear layer, the eye+face combination performs best (AUC: 0.90), followed by eye+head (AUC: 0.82) and face+head (AUC: 0.67) (Figure 8b). The eye+face pairing excels in both methods, leveraging eye gaze and facial features for robust predictions. Table 5 summarizes the best fusion models’ performance, with late fusion models consistently outperforming the intermediate fusion model. The narrower confidence intervals for the late fusion models suggest greater consistency and lower variability.

### 4.3. Fairness Evaluation

Among the best-performing configurations for each input setting, we selected the three best unimodal models and the best three multimodal models based on validation F1 score (MA) to include in our downstream analyses. We evaluated these models for age and gender sensitivity, excluding geographic locations due to insufficient data. We used demographic parity difference (DPD) and equalized odds difference (EOD), where a lower DPD indicates more evenly distributed positive outcomes across groups, while a lower EOD indicates more evenly distributed error rates. To quantify uncertainty in these fairness metrics, we computed 95% confidence intervals using bootstrapping, as described in Section 4.2. It is essential to note that some demographic subgroups—particularly the 2–4 age group and the female subgroup—are represented by relatively few samples, resulting in wider confidence intervals and less stable fairness estimates.

The eye model demonstrates significant variability across age groups (Table 6), performing moderately for the youngest category (2–4 years), with an AUC of 0.69 and a recall of 0.64, suggesting limited reliability for early-age autism identification. Performance improves considerably for older age categories, particularly 9–12, suggesting better effectiveness for older children. The face model exhibits consistent performance across the younger age groups but demonstrates a notable decline in fairness for the 9–12 age group, with an EOD of 0.63. The head model performs well for the age groups 2–4 and 9–12, with an AUC of approximately 0.80, but moderately for the age group 5–8, with an AUC of 0.71 and significant fairness challenges (EOD: 0.63). The late fusion models across the three modalities combine strengths from each unimodal model, leading to improved overall performance and fairness. The late fusion (average) model shows enhanced accuracy and more equitable outcomes across all age groups (DPD: 0.26, EOD: 0.20), reflecting its superior balance between performance and fairness. While the performance improvements are clear and robust, the fairness metrics for late fusion models still exhibit relatively large bootstrap confidence intervals. This indicates that the observed improvements in fairness may not be consistent, and further investigation is needed to establish statistical significance.

Regarding gender, the eye model performs similarly across gender groups (Table 7), showing balanced fairness metrics (DPD: 0.12, EOD: 0.02). In contrast, the face and head models exhibit more pronounced gender disparities, with lower recall for females and larger fairness differences (DPD: 0.29, EOD: 0.42). This indicates that these models are less effective at identifying autism in females, potentially leading to more missed cases compared to males. The late fusion models show improvements in gender performance and good fairness metrics (DPD: 0.20, EOD: 0.08), with narrower confidence intervals. Given the importance of early diagnosis, the late fusion (average) model is the most balanced and fair option for both age and gender groups. Fairness mitigation techniques applied to the head and face models led to marginal improvements, so we focused on the fairer and more effective late fusion methods.

### 4.4. Net Benefit Analysis

**Figure 9.**
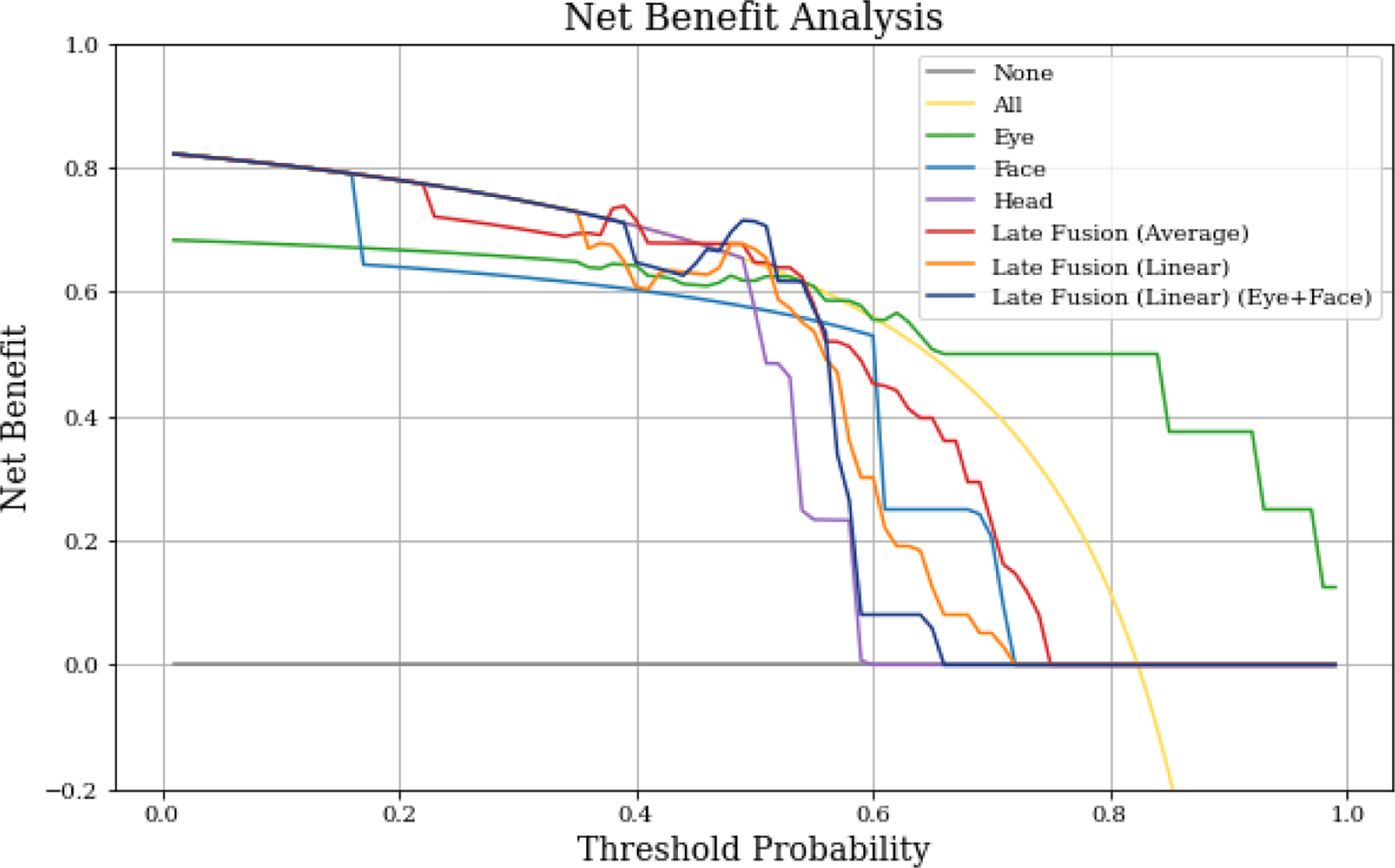
Net benefit analysis. Each line on the graph shows a model’s net benefit (*y*-axis) against threshold values (*x*-axis). The yellow line (“All”) represents treating all as autism, while the grey line (“None”) represents treating none, serving as baselines.

Net benefit analysis (Figure 9) evaluates the clinical utility of each model across a range of decision thresholds. A decision threshold *p*_*t*_ represents the minimum predicted probability at which a clinician would choose to act (e.g., refer a child for further evaluation). Net benefit quantifies how many true positives a model identifies minus the weighted harm of false positives at a given threshold, using the standard decision-curve formula:

$$\text{Net Benefit}=\frac{TP}{n}-\frac{FP}{n}\times \frac{p_{t}}{1-p_{t}}$$

where *p*_*t*_ is the threshold probability and n is the total number of observations.

A model provides clinical value at a given threshold if its net benefit exceeds both trivial strategies, “treat all” and “treat none.” The “treat-all” strategy classifies every child as positive, maximizing sensitivity but producing many false positives. The “treat-none” strategy classifies no child as positive, maximizing specificity but missing all true cases. Net benefit evaluates whether a model achieves a more favorable balance between true positives and false positives than these extremes. Models with higher net benefit therefore support a more reliable and practical diagnostic process, guiding early and timely intervention while efficiently allocating resources.

The late fusion (average) model consistently provides higher net benefits across thresholds up to 0.5, while late fusion (eye+face) performs best around a threshold of 0.5, and the eye-only model surpasses others at thresholds above 0.6. The face and head models show lower net benefits. The dataset’s skewed autism prevalence affects the generalizability of these findings. Given the potential consequences of false positives and false negatives, ethical considerations are essential. As this work is upstream of clinical deployment, any future AI use would aim to support—rather than replace—clinical evaluation and would require caution to mitigate risks such as over-reliance on AI and privacy concerns beyond standard PHI.

## 5. Discussion

This study demonstrates that designing an engineering pipeline and integrating multiple physical indicators into diagnostic models enhances diagnostic precision by generating more informative time-series data and capturing a more comprehensive range of autism-associated signals. This multimodal approach results in models that appear fair, with our observations indicating an equitable and reliable process across diverse demographic cohorts. Further investigation is needed to understand why these models exhibit greater fairness compared to unimodal models. Successfully implementing these predictive models could significantly impact healthcare by optimizing resource allocation, prioritizing children needing further evaluation, and enhancing the efficiency of autism-related services. Additionally, using multimodal time-series data makes the diagnostic process more dynamic, allowing models to adapt to autism’s evolving nature, and thereby supporting effective follow-up care and treatment adjustments. While the present study relied on AWS Rekognition for large-scale feature extraction, we have also implemented on-device face and landmark detection using Google ML Kit [58], which runs in real time on standard smartphones. Given the lightweight size of our behavioral model, these results indicate that end-to-end inference is feasible on mobile devices. The success of these models in autism diagnosis suggests broader potential in other healthcare domains, such as ADHD, highlighting the utility of digital phenotyping and multimodal fusion for improving diagnostics.

Our current models face several limitations. First, camera movement can lead to shifts in the visual field (e.g., the child temporarily leaving the frame). Incorporating device-level accelerometer data and providing real-time user feedback on child distance and identification could mitigate these data drifts, ensuring more reliable predictions. Second, developing an automated preprocessing pipeline is crucial for handling challenges such as detecting when a child is too close or too far from the camera, re-identifying children across frames to handle multiple faces, and maintaining accurate tracking throughout videos. While our filtering and quality-control pipelines ensured high-quality, interpretable inputs, they reduced the dataset to a final sample of 688 videos. This trade-off enhances model robustness but limits statistical power and generalizability, particularly for underrepresented demographic subgroups. A related trade-off exists in our exclusion of “no-face” segments: while prolonged absence of detectable face data often reflects poor video quality and minimal behavioral signal, brief moments when the child turns away or leaves the frame may carry meaningful cues related to social disengagement. Currently, we discard segments with extended face absence, acknowledging the potential loss of useful data.

We emphasize that our results should be interpreted as a proof of concept, and future work will aim to address the aforementioned limitations and expand this study in several directions. Future data collection will explicitly aim for balance and diversity by recruiting participants across multiple community sites, prioritizing underrepresented demographic groups, and incorporating no-ASD children from varied age ranges. The accessibility and scalability of our mobile game provide a unique opportunity to recruit families across diverse socioeconomic and geographic contexts, facilitating inclusive and representative data collection. Additionally, demographic metadata—including age, gender, and skin tone—will be collected to enable fairness auditing and bias correction during model development. In parallel, we plan to integrate the speech modality to capture prosodic and vocal interaction cues that complement facial and head-motion dynamics, enriching the multimodal representation of social behavior. As data collection expands, an age-centric design will enable us to tailor game content and features to different developmental stages, enhancing both engagement and the informativeness of the extracted features. Moreover, prioritizing time-series pretraining will strengthen model robustness. In parallel, developing methods to identify which parts of the sequence contribute most to the model’s decisions will enhance transparency. Together, these steps will make the models more reliable and clinically meaningful. Ultimately, conducting a prospective clinical validation trial will allow for evaluating the reliability and feasibility of the model in real-world diagnostic workflows.

## Figures and Tables

**Table 1.** Number of **children** by demographic category and diagnostic label (ASD vs. no-ASD) in each split.

Demographic	Train		Test		Val	
	ASD	No-ASD	ASD	No-ASD	ASD	No-ASD
	Age					
2–4	19	6	5	3	8	1
5–8	67	10	17	5	19	5
9–12	61	7	24	3	21	2
13–17	1	0	0	0	1	0
	Gender					
Male	114	11	35	5	39	5
Female	34	12	11	6	10	3
	Location					
United States	65	2	25	1	24	0
Outside US	16	0	0	5	4	2
Unknown	67	21	21	5	21	6

**Table 2.** Number of **videos** by demographic category and diagnostic label (ASD vs. no-ASD) in each split.

Demographic	Train		Test		Val	
	ASD	No-ASD	ASD	No-ASD	ASD	No-ASD
	Age					
2–4	39	31	14	6	14	1
5–8	164	24	42	8	42	12
9–12	156	10	56	10	48	6
13–17	4	0	0	0	1	0
	Gender					
Male	279	25	89	13	83	15
Female	84	40	23	11	22	4
	Location					
United States	163	3	59	1	55	0
Outside US	37	0	0	8	8	6
Unknown	163	62	53	15	42	13

**Table 3.** Effects of feature engineering pipeline on model performance. Each model is the best model tuned out of 40 trials, chosen based on validation macro-averaged (MA) F1 score.

Model	AUC Score	F1 Score (MA)
Eye (Raw)	0.66	0.66
Eye (After)	**0.86**	**0.73**
Head (Raw)	0.66	**0.66**
Head (After)	**0.78**	0.63
Face (Raw)	0.63	**0.69**
Face (After)	**0.67**	0.63

Values in **bold** denote the best result per metric for each model (before vs. after feature engineering).

**Table 4.** Performance metrics for the best unimodal model on each modality. The default classification threshold was 0.5. MA denotes macro-average; WA denotes weighted average.

Metric	Eye	Face	Head
AUC Score	**0.86 [0.79, 0.92]**	0.67 [0.55, 0.78]	0.78 [0.69, 0.86]
Accuracy	**0.79 [0.72, 0.86]**	0.75 [0.68, 0.82]	0.75 [0.68, 0.82]
Recall (MA)	**0.84 [0.76, 0.91]**	0.65 [0.55, 0.76]	0.65 [0.55, 0.76]
Recall (WA)	**0.79 [0.72, 0.86]**	0.75 [0.68, 0.82]	0.75 [0.68, 0.82]
Precision (MA)	**0.72 [0.65, 0.79]**	0.62 [0.53, 0.70]	0.62 [0.53, 0.70]
Precision (WA)	**0.89 [0.84, 0.92]**	0.79 [0.71, 0.86]	0.79 [0.71, 0.86]
F1 score (MA)	**0.73 [0.65, 0.81]**	0.63 [0.53, 0.71]	0.63 [0.53, 0.71]
F1 score (WA)	**0.82 [0.75, 0.87]**	0.77 [0.69, 0.83]	0.77 [0.69, 0.83]

MA = macro-average; WA = weighted average. Values in **bold** denote the best score achieved for each metric across models.

**Table 5.** Performance metrics for the best fusion models. The 95% confidence intervals were obtained by bootstrapping the test set. The default classification threshold was 0.5. MA = macro-average, WA = weighted average.

Metric	Late Fusion (Eye, Head, Face) Averaging	Late Fusion (Eye, Head, Face) Linear	Late Fusion (Eye, Face) Linear
AUC Score	**0.90 [0.84, 0.95]**	0.84 [0.77, 0.91]	**0.90 [0.83, 0.95]**
Accuracy	0.82 [0.75, 0.89]	0.84 [0.78, 0.91]	**0.89 [0.83, 0.93]**
Recall (MA)	0.88 [0.81, 0.92]	**0.89 [0.82, 0.94]**	0.85 [0.76, 0.93]
Recall (WA)	0.82 [0.75, 0.89]	0.84 [0.78, 0.91]	**0.89 [0.84, 0.93]**
Precision (MA)	0.75 [0.67, 0.82]	0.76 [0.69, 0.85]	**0.80 [0.72, 0.89]**
Precision (WA)	0.90 [0.87, 0.93]	**0.91 [0.88, 0.94]**	0.90 [0.85, 0.94]
F1 score (MA)	0.77 [0.69, 0.85]	0.79 [0.71, 0.88]	**0.82 [0.74, 0.90]**
F1 score (WA)	0.84 [0.78, 0.90]	0.86 [0.80, 0.92]	**0.89 [0.83, 0.93]**

MA = macro-average; WA = weighted average. Values in **bold** denote the best score achieved for each metric across models.

**Table 6.** Summary of model fairness across age groups with confidence intervals. The sample sizes are as follows: 2–4 years (20), 5–8 years (50), and 9–12 years (66).

Model	Age	Accuracy	Recall	Precision	AUC Score	F1 Score	Demographic Parity Diff.	Equalized Odds Diff.
Eye Model	2–4	0.70 [0.50, 0.90]	0.64 [0.38, 0.87]	0.90 [0.67, 1.00]	0.69 [0.44, 0.91]	0.75 [0.52, 0.92]	0.21 [0.05, 0.48]	0.20 [0.10, 0.60]
	5–8	0.74 [0.62, 0.86]	0.71 [0.58, 0.85]	0.97 [0.89, 1.00]	0.81 [0.69, 0.91]	0.82 [0.72, 0.91]		
	9–12	0.86 [0.77, 0.94]	0.84 [0.74, 0.93]	1.00 [1.00, 1.00]	0.94 [0.87, 0.99]	0.91 [0.85, 0.96]		
Face Model	2–4	0.80 [0.60, 0.95]	0.79 [0.56, 1.00]	0.92 [0.73, 1.00]	0.79 [0.45, 1.00]	0.85 [0.64, 0.97]	0.23 [0.02, 0.32]	0.63 [0.27, 1.00]
	5–8	0.74 [0.62, 0.86]	0.76 [0.62, 0.88]	0.91 [0.81, 1.00]	0.75 [0.52, 0.94]	0.83 [0.73, 0.91]		
	9–12	0.74 [0.64, 0.83]	0.84 [0.74, 0.93]	0.85 [0.75, 0.94]	0.55 [0.37, 0.72]	0.85 [0.77, 0.91]		
Head Model	2–4	0.80 [0.60, 0.95]	0.79 [0.54, 1.00]	0.92 [0.75, 1.00]	0.82 [0.61, 0.98]	0.85 [0.67, 0.97]	0.23 [0.03, 0.34]	0.63 [0.27, 1.00]
	5–8	0.74 [0.60, 0.86]	0.76 [0.63, 0.88]	0.91 [0.81, 1.00]	0.71 [0.49, 0.89]	0.83 [0.73, 0.92]		
	9–12	0.74 [0.64, 0.83]	0.84 [0.74, 0.93]	0.85 [0.75, 0.94]	0.80 [0.68, 0.90]	0.85 [0.76, 0.91]		
Late Fusion (Average)	2–4	0.75 [0.55, 0.90]	0.64 [0.33, 0.87]	1.00 [1.00, 1.00]	0.93 [0.77, 1.00]	0.78 [0.56, 0.93]	0.26 [0.04, 0.50]	0.20 [0.07, 0.53]
	5–8	0.80 [0.68, 0.90]	0.79 [0.66, 0.90]	0.97 [0.90, 1.00]	0.84 [0.69, 0.95]	0.87 [0.77, 0.94]		
	9–12	0.86 [0.77, 0.94]	0.84 [0.74, 0.93]	1.00 [1.00, 1.00]	0.93 [0.85, 0.98]	0.91 [0.85, 0.96]		
Late Fusion (Linear)	2–4	0.75 [0.55, 0.90]	0.64 [0.38, 0.89]	1.00 [1.00, 1.00]	0.64 [0.40, 0.88]	0.78 [0.55, 0.94]	0.27 [0.04, 0.51]	0.21 [0.07, 0.53]
	5–8	0.84 [0.74, 0.94]	0.83 [0.71, 0.95]	0.97 [0.90, 1.00]	0.85 [0.73, 0.94]	0.90 [0.82, 0.96]		
	9–12	0.88 [0.79, 0.95]	0.86 [0.76, 0.95]	1.00 [1.00, 1.00]	0.90 [0.81, 0.96]	0.92 [0.86, 0.97]		
Late Fusion (Linear, Eye+Face)	2–4	0.95 [0.85, 1.00]	0.93 [0.77, 1.00]	1.00 [1.00, 1.00]	0.93 [0.79, 1.00]	0.96 [0.87, 1.00]	0.17 [0.02, 0.22]	0.38 [0.14, 0.75]
	5–8	0.84 [0.74, 0.94]	0.88 [0.78, 0.98]	0.93 [0.83, 1.00]	0.85 [0.71, 0.96]	0.90 [0.83, 0.96]		
	9–12	0.91 [0.83, 0.97]	0.93 [0.85, 0.98]	0.96 [0.91, 1.00]	0.93 [0.85, 0.98]	0.95 [0.90, 0.98]		

**Table 7.** Summary of model fairness across gender groups with 95% confidence intervals. Sample sizes: female (34), male (102).

Model	Gender Group	Accuracy	Recall	Precision	AUC Score	F1 Score	Demographic Parity Diff.	Equalized Odds Diff.
Eye Model	Female	0.82 [0.68, 0.94]	0.78 [0.60, 0.95]	0.95 [0.83, 1.00]	0.81 [0.65, 0.95]	0.86 [0.72, 0.96]	0.12 [0.00, 0.22]	0.02 [0.01, 0.32]
	Male	0.78 [0.69, 0.86]	0.76 [0.66, 0.84]	0.99 [0.95, 1.00]	0.87 [0.78, 0.93]	0.86 [0.80, 0.91]		
Face Model	Female	0.68 [0.53, 0.82]	0.65 [0.46, 0.84]	0.83 [0.63, 1.00]	0.67 [0.44, 0.88]	0.73 [0.56, 0.86]	0.29 [0.02, 0.41]	0.42 [0.16, 0.75]
	Male	0.77 [0.69, 0.85]	0.84 [0.76, 0.91]	0.89 [0.83, 0.95]	0.63 [0.48, 0.78]	0.86 [0.81, 0.92]		
Head Model	Female	0.68 [0.53, 0.82]	0.65 [0.45, 0.85]	0.83 [0.65, 1.00]	0.70 [0.52, 0.86]	0.73 [0.57, 0.86]	0.29 [0.02, 0.41]	0.42 [0.14, 0.78]
	Male	0.77 [0.68, 0.85]	0.84 [0.76, 0.91]	0.89 [0.82, 0.95]	0.76 [0.61, 0.88]	0.86 [0.80, 0.91]		
Late Fusion (Average)	Female	0.82 [0.68, 0.94]	0.74 [0.55, 0.90]	1.00 [1.00, 1.00]	0.83 [0.65, 0.95]	0.85 [0.71, 0.96]	0.21 [0.00, 0.27]	0.08 [0.01, 0.30]
	Male	0.82 [0.74, 0.90]	0.81 [0.72, 0.89]	0.99 [0.96, 1.00]	0.91 [0.83, 0.97]	0.88 [0.83, 0.93]		
Late Fusion (Linear)	Female	0.85 [0.74, 0.97]	0.78 [0.60, 0.93]	1.00 [1.00, 1.00]	0.79 [0.62, 0.94]	0.88 [0.76, 0.97]	0.22 [0.01, 0.25]	0.08 [0.01, 0.30]
	Male	0.84 [0.77, 0.91]	0.83 [0.74, 0.90]	0.99 [0.96, 1.00]	0.87 [0.76, 0.91]	0.90 [0.85, 0.94]		
Late Fusion (Linear, Eye+Face)	Female	0.82 [0.68, 0.94]	0.83 [0.65, 0.96]	0.90 [0.76, 1.00]	0.82 [0.65, 0.96]	0.86 [0.73, 0.96]	0.21 [0.01, 0.28]	0.11 [0.04, 0.39]
	Male	0.91 [0.85, 0.96]	0.93 [0.87, 0.98]	0.96 [0.92, 1.00]	0.85 [0.81, 0.97]	0.95 [0.91, 0.98]		

**Figure A2.**
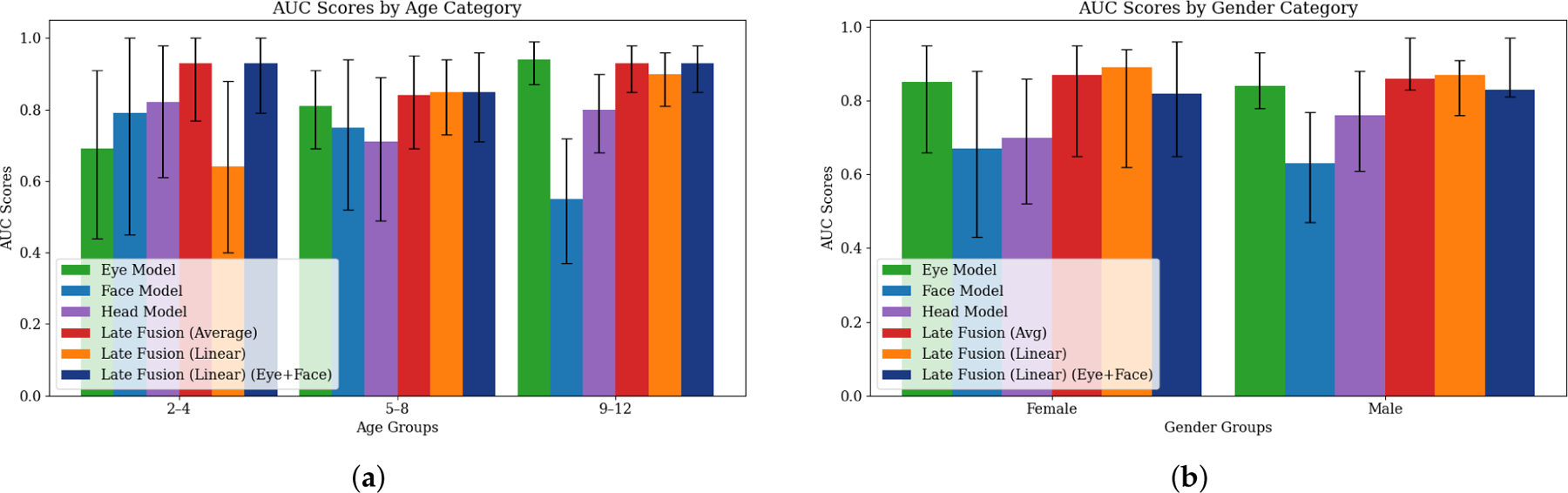

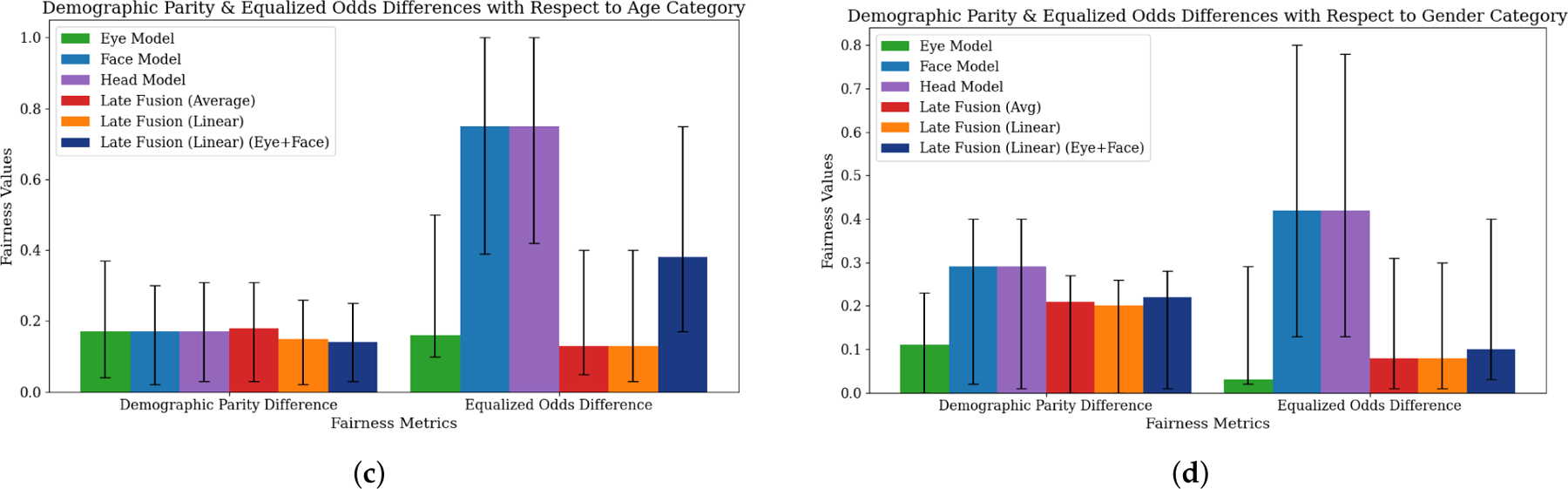
AUC and fairness metrics across age and gender: (**a**) AUC scores by age category and model. (**b**) AUC scores by gender category and model. (**c**) Demographic parity and equalized odds differences by age category. (**d**) Demographic parity and equalized odds differences by gender category.

## Data Availability

The datasets analyzed during the current study are not publicly available due to the inclusion of Protected Health Information (PHI). Researchers who wish to gain access may email the corresponding author to request access under a Data Use Agreement with Stanford University.

## Appendix A

**Table A1.** Filtering thresholds based on the visual features extracted using AWS for each video. Through manual inspection of a diverse subset of samples, we identified minimum sharpness and brightness levels below which even human reviewers could not reliably extract gaze, head pose, or facial features. These criteria constitute the conditions for a video to be considered.

Filtering Criteria	Variables	Thresholds
Quality	Sharpness, brightness	Sharpness (0–100) > 4; Brightness (0–100) > 20
Face Detection	No face proportion, Multi-face proportion, Face size	No face detection (0–1) < 0.6; Multi-face detection (0–1) < 0.3; Face size (0–100) > 0.01
Eye Visibility	Head pose (pitch, roll, yaw), Eyes' confidence	Pitch (−180–180) < 45; Roll (−180–180) < 45; Yaw (−180–180) < 45; Eyes' confidence (0–100) > 75

**Table A2.** Hyperparameter search space for unimodal models.

Hyperparameter	Range Searched
Model	LSTM, GRU, CNN+LSTM, CNN+GRU
Hidden Size (of LSTM/GRU)	{16, 32, 64}
Batch Size	{32, 48, 64, 100}
Number of Layers	[4, 8]
Dropout Probability	[0.1, 0.3]
Learning Rate	[1 × 10^−4^, 1 × 10^−1^]
Weight Decay	[1 × 10^−5^, 1 × 10^−2^]
Optimizer	Adam
Loss Function	Cross-entropy, focal loss

**Table A3.** Hyperparameter search space for intermediate fusion models.

Hyperparameter	Range Searched
Batch Size	{16, 32, 64}
Learning Rate	[1 × 10^−4^, 1 × 10^−1^]
First Hidden Size	{128, 192, 256}
Second Hidden Size	{32, 64, 128}
Third Hidden Size	{32, 64}
Optimizer	Adam
Loss Function	Cross-entropy

**Table A4.** Best hyperparameters for the unimodal models.

Hyperparameter	Eye Gazing	Head Pose	Facial Landmarks
Model	LSTM	LSTM	LSTM
Hidden Size	64	32	48
Batch Size	64	48	48
Number of Layers	8	4	4
DropoutProbability	0.27	0.19	0.18
Learning Rate	0.03	0.0003	0.05
Weight Decay	1 × 10^−5^	4 × 10^−5^	4 × 10^−5^
Optimizer	Adam	Adam	Adam
Loss Function	Cross-Entropy	Cross-Entropy	Cross-Entropy

**Table A5.** Effect of upsampling the no-ASD class in the training set on model performance. Upsampling duplicated up to two additional videos per no-ASD child when available, increasing the number of no-ASD training videos from 65 to 97. Each model is the best of 40 trials, selected by validation macro-averaged F1.

Model	AUC Score	F1 Score (MA)
Eye (No Upsampling)	**0.86**	**0.73**
Eye (Upsampling)	0.76	0.62
Head (No Upsampling)	**0.78**	**0.63**
Head (Upsampling)	0.60	0.56
Face (No Upsampling)	0.67	**0.63**
Face (Upsampling)	**0.76**	0.61

MA = macro-average. Values in **bold** denote the best result per metric (before vs. after upsampling).

**Table A6.** Best hyperparameters for intermediate fusion.

Hyperparameter	Value
Batch Size	16
Learning Rate	0.07
First Hidden Size	256
Second Hidden Size	32
Third Hidden Size	64
Number of Epochs	15

**Table A7.** Best hyperparameters for late fusion (linear).

Hyperparameter	Value
Batch Size	32
Learning Rate	5 × 10^−4^
Number of Epochs	11

## Abbreviations

The following abbreviations are used in this manuscript:

ASD: Autism Spectrum Disorder
MA: Macro-averaged
WA: Weighted-averaged
